# CROSS-NATIONAL COMPARISONS OF STRESS AND WELL-BEING IN THE INTERNATIONAL FAMILY OF HEALTH AND RETIREMENT STUDIES

**DOI:** 10.1093/geroni/igac059.2656

**Published:** 2022-12-20

**Authors:** Yoobin Park, Alexandra Crosswell, Drystan Phillips

**Affiliations:** University of California, San Francisco, San Francisco, California, United States; University of California, San Francisco, San Francisco, California, United States; University of Southern California, Los Angeles, California, United States

## Abstract

Strong evidence demonstrates the long-term influence of stress and well-being on psychological, social, and physical health outcomes across the lifespan. Because of this, stress and well-being measures have been added to nearly all of the International Family of Health and Retirement Studies. However, this newly available data has not been compared cross-nationally or within-country to unpack how culture influences these important predictors of healthy aging. Using the Gateway to Global Aging Data, which provides harmonized data from the Health and Retirement Study and its sibling nationally representative studies, levels of self-reported stress (e.g. job stress, discrimination, loneliness) and well-being (e.g. quality of life, life satisfaction) are compared across 30 countries. Data come from the following studies: HRS, ELSA, SHARE, TILDA, CHARLS, KLoSA, MHAS, and JSTAR. We used data from the latest study wave for which the relevant survey was implemented. Average age of participants across studies is 67 and 55% are women. Initial analyses show stressor specific findings such as participants in Korea reported greater work stress than participants in Japan, England, the United States, and across Europe, and the United States reported higher loneliness than China and England, but not higher than Ireland. Reporting cross-national and within-country variation in these measures will be generative in pointing to new research directions for understanding how culture influences health and aging trajectories.

